# Accuracy of Canadian CT Head Rule and New Orleans Criteria for Minor Head Trauma; a Systematic Review and Meta-Analysis

**Published:** 2020-09-08

**Authors:** Abeer Kadum Abass Alzuhairy

**Affiliations:** Lecturer in Diagnostic Imaging - Surgery Department, College of Medicine, University of Sulimani Kurdistan Region, Iraq.

**Keywords:** Sensitivity and Specificity, Predictive Value of Tests, Craniocerebral Trauma, Systematic Review, Meta-Analysis

## Abstract

**Introduction:**

The present meta-analysis has two objectives; primarily, the predictive values of Canadian computed tomography (CT) head rule (CCHR) and New Orleans Criteria (NOC) will be compared. Secondly, the possibility of interchangeable use of the two models in cases of contraindication will be evaluated.

**Methods:**

An extensive search was performed in Medline, Embase, Scopus and Web of Science electronic databases from the inception of databases until the end of July 2020. All prospective and retrospective observational and diagnostic accuracy studies comparing NOC and CCHR on a single group of patients were included. Data were entered to STATA 14.0 statistical program, and analyses were performed using “*metandi*” command.

**Results:**

Data from 14 articles were included (21140 samples). Summary sensitivity, specificity, and diagnostic odds ratio of CCHR in prediction of positive CT findings were 89.8% (95% CI: 79.6 to 95.2), 38.3% (95% CI: 34.0 to 42.8), and 5.5 (95% CI: 2.3 to 13.1), respectively. In addition, summary sensitivity, specificity, and diagnostic odds ratio of NOC in prediction of positive CT findings were 97.2% (95% CI: 89.7 to 99.2), 12.3% (95% CI: 7.4 to 19.8), and 4.8 (95% CI: 1.2 to 18.3), respectively. Summary sensitivity, specificity, and diagnostic odds ratio of CCHR in prediction of clinically important TBI (ciTBI) in mild TBI patients were 92.5% (95% CI: 79.5 to 97.5), 40.1% (95% CI: 34.8 to 45.6), and 8.3 (95% CI: 2.4 to 29.2), respectively. In addition, summary sensitivity, specificity, and diagnostic odds ratio of NOC in prediction of ciTBI were 98.3% (95% CI: 93.8 to 99.6), 8.5% (95% CI: 4.8 to 14.5), and 5.4 (95% CI: 1.5 to 20.0), respectively.

**Conclusion:**

The present meta-analysis demonstrated that both CCHR and NOC scores have a good predictive value in predicting the presence of abnormal findings in CT scan and ciTBI. The similar performance of CCHR and NOC models results in their interchangeable use in cases of contraindication.

## Introduction

Traumatic brain injury (TBI) is one of the most common causes of emergency department referrals worldwide. The global burden of TBI has been increasing in recent years, with an increasing prevalence rate of 8.4% between 1990 and 2016, accompanied by a increasing incidence rate of 3.6% in years of life lived with disability (1). 

The gold standard in diagnosis of intracranial complications after TBI is computed tomography (CT) scan. However, mild TBI is the most prevalent type of TBI, in which brain CT scans are usually normal (2). Therefore, unnecessary CT scans are quite prevalent. In an attempt to reduce the number of unnecessary CT scans, several scoring systems have been introduced (3-10), including the Canadian CT head rule (CCHR) and the New Orleans Criteria (NOC) (11, 12). The performance of these two models has been validated in various studies (13-16), but limitations have been attributed to each of these scoring systems. For instance, NOC is only applicable in patients with a Glasgow Coma Scale (GCS) of 15, and cannot be used for patients with a GCS of 14 or 13. On the other hand, CCHR cannot be applied to patients under 18 years of age, patients on blood thinners and patients having seizures after a head trauma.

These limitations have caused an uncertainty regarding which of the two scoring systems has better performance in identifying high-risk patients, and whether these tools can be used interchangeably. To evaluate the performance of a rule out criteria in identifying high risk patients in mild TBI, two major outcomes will be assessed, including positive CT scan findings and clinically important TBI (ciTBI). 

Existing studies have attempted to evaluate the diagnostic value of these two decision rules in predicting positive CT findings and ciTBI to reduce unnecessary CT scans (13, 17-20). However, a consensus over the subject is yet to be achieved. Hence, the present meta-analysis has two objectives; primarily, the predictive values of CCHR and NOC will be compared; and secondly, the possibility of interchangeable use of the two models in cases of contraindication will be evaluated.

## Methods

**Search strategy**

To achieve the objectives of the present study, an extensive search was performed in Medline, (via PubMed), Embase, Scopus and Web of Science electronic databases from the inception of databases until the end of July 2020. In addition, PubMed Central records were also added, so that no articles would be missed. Initially, keywords related to traumatic brain injury in combination with keywords related to Canadian CT head rule or New Orleans Criteria were used in the search. However, the number of the achieved records was low. Hence, to perform a more extensive search, only keywords related to Canadian CT head rule or New Orleans Criteria were used in the search. The search term in Medline database is presented below.

"Canadian computed tomography head rule" [Title / Abstract] OR "Canadian CT head rule" [Title / Abstract] OR "New Orleans criteria" [Title / Abstract]

In addition to the systematic search, a manual search was also performed via Google and Google scholar search engines and in related articles’ bibliography to include articles that were not indexed or not found.

**Selection criteria**

All prospective and retrospective observational and diagnostic accuracy studies, performing a comparison between the two models of NOC and CCHR on a single group of patients, were entered to the present study. Exclusion criteria were lack of reporting sensitivity and specificity or true positive, true negative, false positive and false negative. Other exclusion criteria were lack of assessment of CT scan findings or ciTBI, studies performed on children, failure to compare CCHR and NOC criteria and review studies.

**Data extraction and quality assessment**

Two independent researchers screened the titles and abstracts of the retrieved records, based on the inclusion and exclusion criteria. Next, potentially relevant studies were screened more carefully, and finally, related articles were included. Then, the two researchers independently summarized the included articles into a checklist, composed of data including first author’s name, publication year, country, study design, sampling method, sample size, age, gender distribution, outcome, sensitivity, specificity, true positive, true negative, false positive and false negative. Any disagreements were resolved by discussion. Furthermore, evaluated outcomes included positive findings on CT and ciTBI. Quality control of the studies was performed based on the Quality Assessment of Diagnostic Accuracy Studies version 2.0 (QUADAS-2) (21).

**Statistical analysis**

Data were entered to STATA 14.0 statistical program, and analyses were performed using “metandi” command. Analyses were performed in two separate sections according to the evaluated outcomes. Initially, the predictive values of CCHR and NOC in CT scan positive findings were assessed. Next, the predictive values of CCHR and NOC in ciTBI were evaluated. To assess publication bias, Deek’s funnel plot asymmetry test was used. Results are presented as sensitivity, specificity, positive and negative likelihood ratios, and diagnostic odds ratio with 95% confidence interval (95% CI).

## Results

**Characteristics**

**Figure 1:**
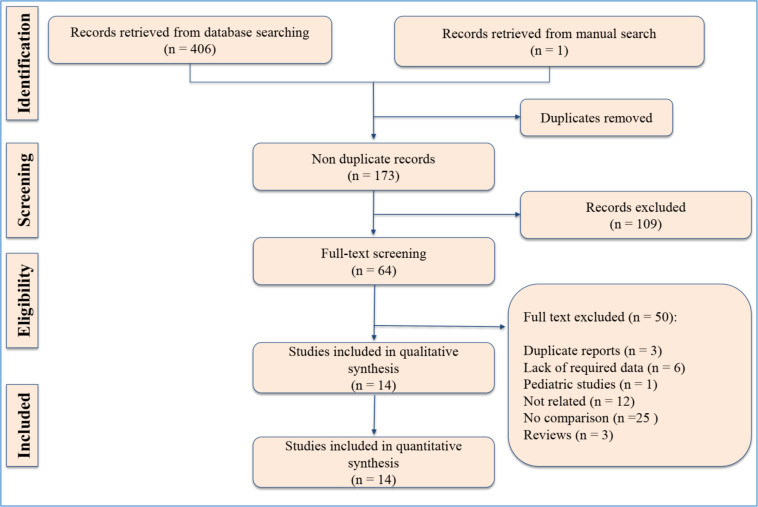
PRISMA flow diagram of the present meta-analysis

The search resulted in 406 records. After eliminating duplicates and screening, 64 titles of potentially relevant studies were screened in more detail, and finally data from 14 articles were included in the present meta-analysis (11, 13, 14, 17-19, 22-29) (Figure 1). These articles entailed 21140 samples. Of these, 1940 patients had a positive CT scan finding and 19180 patients had a negative CT scan. Of the 1940 patients with positive CT scan, 594 ciTBIs were observed. The evaluated outcome was only positive CT scan findings in eight articles, only ciTBI in two studies, and both outcomes in 5 studies. Table 1 summarizes the characteristics of the included studies.

**Accuracy of CCHR and NOC in prediction of positive CT findings**

**Figure 2:**
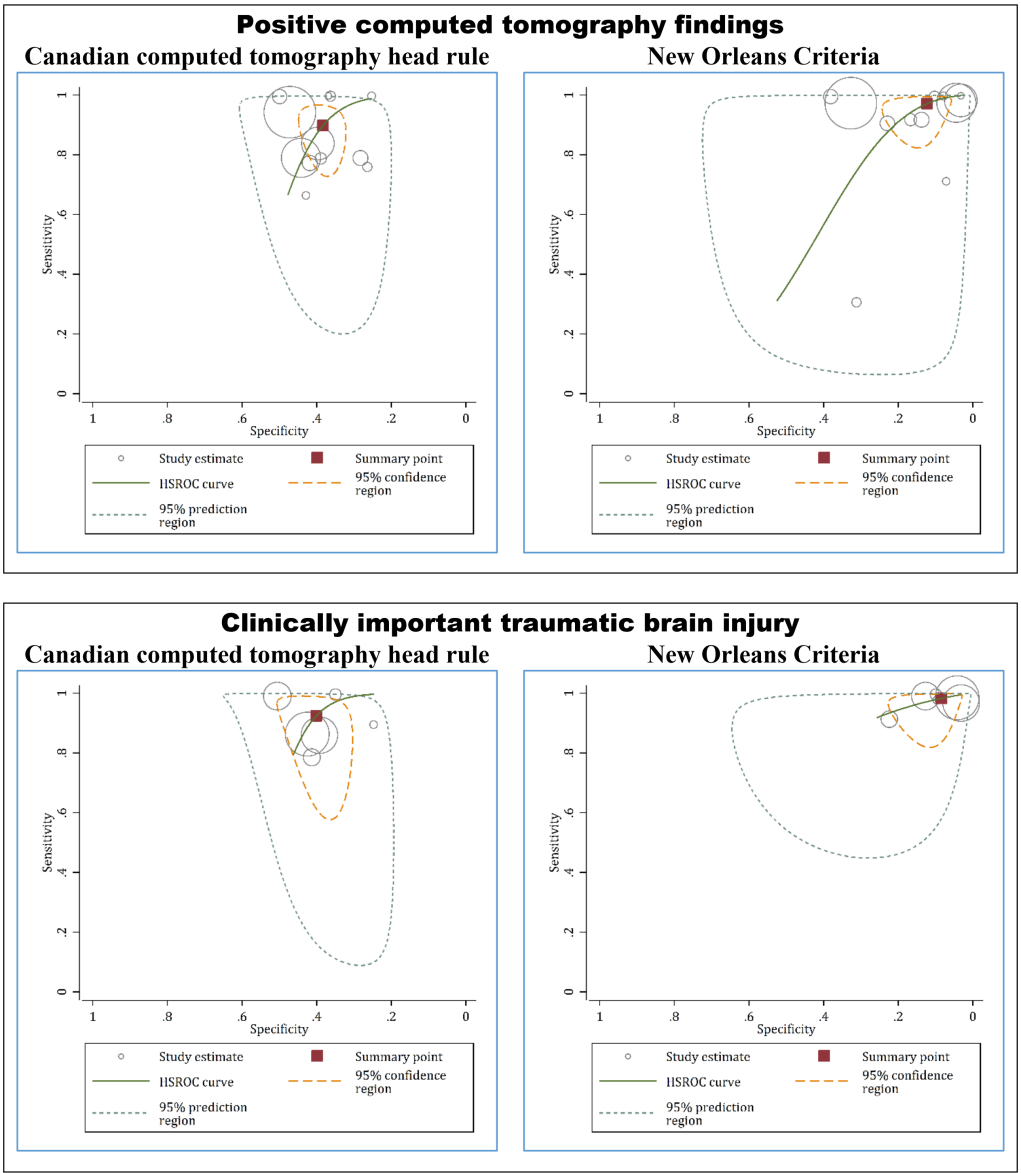
Hierarchical summary receiver-operating characteristic (HSROC) curves of Canadian computed tomography head rule and New Orleans criteria in prediction of computed tomography findings and clinically important traumatic brain injury.

Summary sensitivity, specificity, and diagnostic odds ratio of CCHR in prediction of positive CT findings were 89.8% (95% CI: 79.6 to 95.2), 38.3% (95% CI: 34.0 to 42.8), and 5.5 (95% 2.3 to 13.1), respectively. In addition, summary sensitivity, specificity, and diagnostic odds ratio of NOC in prediction of positive CT findings were 97.2% (95% CI: 89.7 to 99.2), 12.3% (95% CI: 7.4 to 19.8), and 4.8 (95% CI: 1.2 to 18.3), respectively (Table 2 and Figure 2).

**Accuracy of CCHR and NOC in prediction of ciTBI**

 Summary sensitivity, specificity, and diagnostic odds ratio of CCHR in prediction of ciTBI in patients with mild traumatic brain injuries were 92.5% (95% CI: 79.5 to 97.5), 40.1% (95% CI: 34.8 to 45.6), and 8.3 (95% CI: 2.4 to 29.2), respectively. In addition, summary sensitivity, specificity, and diagnostic odds ratio of NOC in prediction of ciTBI were 98.3% (95% CI: 93.8 to 99.6), and 8.5% (95% CI: 4.8 to 14.5), 5.4 (95% CI: 1.5 to 20.0), respectively (Table 2 and Figure 2).

**Quality assessment and risk of bias**

**Figure 3:**
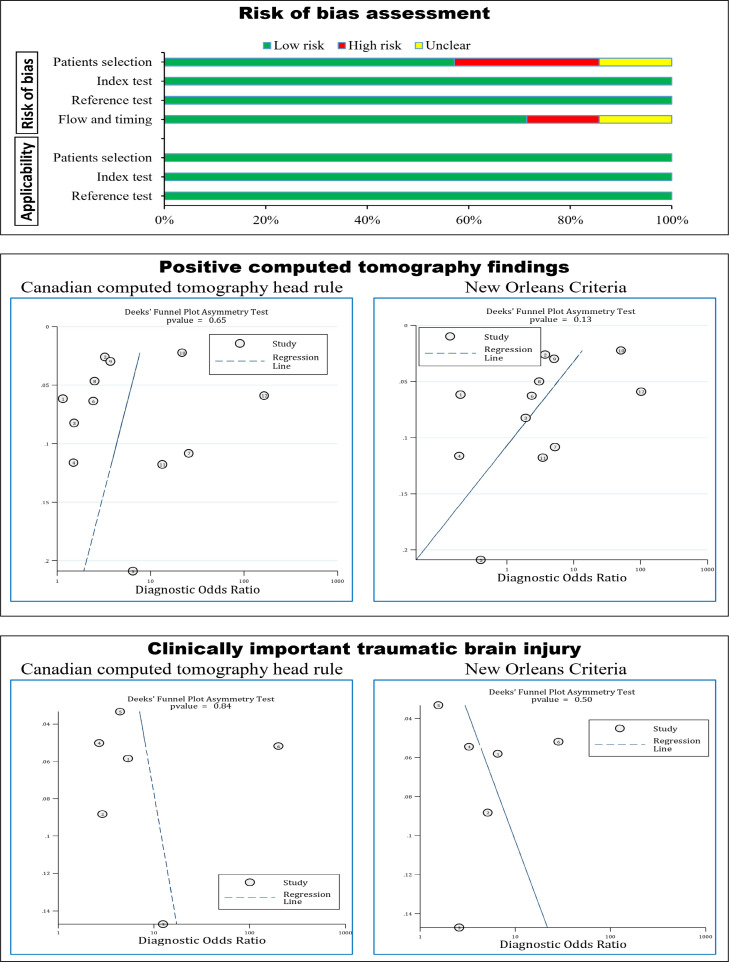
Risk of bias assessment and publication bias in Canadian computed tomography head rule and New Orleans criteria according to outcome.

Risk of bias assessment based on QUADAS-2 scale showed that risk of bias regarding patient selection was high in four studies, and unclear in 2 studies. In addition, flow and timing was high risk in 2 studies and unclear 2 other studies. Other items were categorized as low risk in risk of bias assessment and applicability domains (Table 3 and figure 3).

Deeks’ funnel asymmetry plots showed that there was no publication bias in assessment of accuracy of CCHR and NOC in prediction of positive CT findings (p _for CCHR_ = 0.65; p _for NOC_ = 0.13) and ciTBI (p _for CCHR_ = 0.84; p _for NOC_ = 0.50) (Figure 3).

## Discussion

The present meta-analysis showed that both CCHR and NOC scores have good predictive value in predicting abnormal findings in CT scan and ciTBI. Although NOC’s sensitivity was slightly higher than that of CCHR, but CCHR’s specificity was much higher than that of NOC. However, in interpreting the results of the two models, bear in mind that both models are designed for screening patients and identifying high risk ones to decrease the number of unnecessary CT scans. In screening tests, sensitivity is more important than specificity, because the task of these tests is to select eligible patients for CT scan. Hence, these two models are not designed for a definite diagnosis determination, and a low specificity is not considered a weakness of the tests. Accordingly, it can be concluded that the values of both CCHR and NOC models in screening minor head trauma patients are similar.

**Table 1:** Characteristics of the included studies

**Author; year; country**	**Design**	**Sampling method**	**Total sample**	**Total age**	**Male number**	**Outcome**
Chobdari; 2018; Iran	Prospective observational	Convenience	264	>14	211	Positive CT
Foks; 2018; Netherlands	Prospective observational	Consecutive	4557	16 to 101	2656	Positive CT; ciTBI
Jones; 2020; USA	Prospective observational	Consecutive	679	>=16	420	Positive CT
Kavalci; 2014; Turkey	Prospective observational	NR	175	>=18	106	Positive CT
Korley; 2013; USA	Prospective observational	Convenience	130	>=14	63	Positive CT
Lo; 2016; Hong Kong	Retrospective observational	Consecutive	383	All ages	NR	Positive CT
Mata-Mbemba; 2016; Japan	Prospective observational	Consecutive	142	17 to 88	96	ciTBI
Papa; 2012; USA	Prospective observational	Consecutive	314	18 to 89	201	Positive CT; ciTBI
Ro; 2011; Korea	Prospective observational	Consecutive	696	46.1+18.9	447	Positive CT; ciTBI
Smith; 2005; Netherlands	Prospective observational	Consecutive	3181	16 to 102	2244	Positive CT; ciTBI
Stein; 2009; USA	Prospective observational	Consecutive	7955	>10	4415	Positive CT
Stiell; 2005; Canada	Prospective observational	Consecutive	1822	16 to 99	1246	ciTBI
Valle Alonso; 2016; Spain	Prospective observational	NR	217	16 to 102	135	Positive CT
Yang; 2017; China	Retrospective observational	Consecutive	625	>18	339	Positive CT

ciTBI: Clinically important traumatic brain injuries; CT: Computed tomography; NR: Not reported.

**Table 2:** Prognostic performance of Canadian computed tomography head rule and New Orleans criteria according to outcome

	**Positive CT **		**ciTBI**	
	Value	95% CI	Value	95% CI
**Canadian computed tomography head rule**				
True positive	1554	---	524	---
True negative	7576	---	4300	---
False Positive	9782	---	5818	---
False negative	244	---	70	---
Sensitivity	89.8	79.6 - 95.2	92.5	79.5 - 97.5
Specificity	38.3	34.1 - 42.8	40.1	34.8 - 45.6
Positive likelihood ratio	1.5	1.3 - 1.6	1.5	1.3 - 1.8
Negative likelihood ratio	0.3	0.1 - 0.6	0.2	0.1 - 0.6
Diagnostic odds ratio	5.5	2.3 - 13.1	8.3	2.4 - 29.2
				
**New Orleans criteria**				
True positive	1643	---	562	---
True negative	3278	---	664	---
False Positive	14109	---	9436	---
False negative	134	---	14	---
Sensitivity	97.2	89.7 - 99.3	98.3	93.8 - 99.6
Specificity	12.3	7.4 - 19.8	8.5	4.8 - 14.5
Positive likelihood ratio	1.1	1.0 - 1.2	1.1	1.0 - 1.1
Negative likelihood ratio	0.2	0.1 - 0.8	0.2	0.1 - 0.7
Diagnostic odds ratio	4.8	1.3 - 18.3	5.4	1.5 - 20.0

CI: Confidence interval; ciTBI: Clinically important traumatic brain injuries; CT: Computed tomography.

**Table 3:** Risk of bias assessment of included studies

Author; Year	**Risk of bias**				**Applicability**		
	**Patients selection**	**Index test**	**Reference test**	**Flow and timing**	**Patients selection**	**Index test**	**Reference test**
Chobdari; 2018	High	Low	Low	High	Low	Low	Low
Foks; 2018	Low	Low	Low	Low	Low	Low	Low
Jones; 2020	Low	Low	Low	Low	Low	Low	Low
Kavalci; 2014	Unclear	Low	Low	Low	Low	Low	Low
Korley; 2013	High	Low	Low	High	Low	Low	Low
Lo; 2016	High	Low	Low	Unclear	Low	Low	Low
Mata-Mbemba; 2016	Low	Low	Low	Low	Low	Low	Low
Papa; 2012	Low	Low	Low	Low	Low	Low	Low
Ro; 2011	Low	Low	Low	Low	Low	Low	Low
Smith; 2005	Low	Low	Low	Low	Low	Low	Low
Stein; 2009	Low	Low	Low	Low	Low	Low	Low
Stiell; 2005	Low	Low	Low	Low	Low	Low	Low
Valle alonso; 2016	Unclear	Low	Low	Low	Low	Low	Low
Yang; 2017	High	Low	Low	Unclear	Low	Low	Low

Low: Low risk; High: High risk; Unclear: Unclear risk of bias.

The similarity of the two models in predicting positive CT findings and ciTBI is a profound finding in the present study, because both CCHR and NOC models have limitations that affect their performance. For instance, there is no indication for using NOC in patients with a GCS less than 15, while CCHR is recommended for patients having a GCS between 13 and 15. On the other hand, patients under the age of 18 years, patients on blood thinners and patients with seizures after head traumas are the exclusion criteria for CCHR. As a result, in cases of contraindication, the other score can be used instead. 

In a similar systematic review in 2017, Webster et al. showed that both CCHR and NOC models have the same sensitivity in predicting ciTBI, while the specificity of CCHR is much higher than that of NOC (10). The findings of the current study are also in line with that of Webster et al. However, in the present article, more studies were included and at the end, a meta-analysis was performed, unlike in the review by Webster et al.

The quality control was performed according to QUADAS-2 instructions, showing an acceptable status regarding most of the included studies in the fields of risk of bias and applicability, so the results of the present meta-analysis are authentic. On the other hand, no publication bias was observed in the present study, confirming the validity of the findings of our study.

The current study demonstrated that the values of CCHR and NOC models in predicting positive CT findings and ciTBI are desirable. However, in addition to the diagnostic value, cost-effectiveness is also important. For this purpose, a meta-analysis evaluating 24 economic studies showed that CCHR is an economically attractive tool in management of mild TBI (30). 

Nevertheless, there existed limitations in the present study. One of these limitations was the lack of real-time score assessments in most of the included studies. The studies were designed in a way that in most of them, risk factors for intracranial complications used in the CCHR and NOC were collected and then their performance was evaluated. While, it was more desirable if the physician or the technician would have determined and recorded the decision whether to perform a CT scan based on the two criteria of NOC and CCHR, using a decision tree, and eventually compared the CT findings with their decision. Hence, it is recommended that in future studies, real-time value of both models in predicting positive CT findings and ciTBI be compared.

## Conclusion

The present meta-analysis demonstrated that both CCHR and NOC scores have a good predictive value in predicting the presence of abnormal findings in CT scan and ciTBI. The similar performance of CCHR and NOC models results in their interchangeable use in cases of contraindication.
